# Pancreatitis After Treatment With Encorafenib, Binimetinib, and Cetuximab for BRAF V600E Mutation-Positive Colorectal Cancer

**DOI:** 10.7759/cureus.60188

**Published:** 2024-05-13

**Authors:** Yuika Kureyama, Yutaka Hanaoka, Daisuke Tomita, Shuichiro Matoba, Hiroya Kuroyanagi

**Affiliations:** 1 Department of Colorectal Surgery, Toranomon Hospital, Tokyo, JPN

**Keywords:** metastasis, cetuximab, binimetinib, encorafenib, pancreatitis, colorectal cancer, braf v600e mutation

## Abstract

BRAF V600E mutation-positive advanced recurrent colorectal cancer has a poor prognosis. Encorafenib, binimetinib, and cetuximab were approved for use to treat this cancer in 2020 in Japan. Here, we present the case of a patient with BRAF V600E mutation-positive colorectal cancer, who was treated with encorafenib, binimetinib, and cetuximab, and developed grade 3 pancreatitis at our hospital. After pancreatitis treatment, the drug doses were reduced from 300 mg to 225 mg of encorafenib and from 90 mg to 60 mg of binimetinib, and the treatment was resumed. Since then, no grade 3 or higher adverse events were observed. Although pancreatitis has been reported to occur after the use of encorafenib and binimetinib, it is rare. With appropriate dose reduction and attention to side effects, this regimen is considered feasible for the long-term treatment of BRAF V600E mutation-positive advanced recurrent colorectal cancer in patients aged >70 years.

## Introduction

While BRAF mutation-positive advanced recurrent colorectal cancer has a very poor prognosis [1], it is also infrequent, accounting for approximately 8% of all cases [2]. FOLFOXIRI (folinic acid, 5-fluorouracil, oxaliplatin, and irinotecan) therapy is the only regimen that has shown promise for treatment, but based on the results of the 2019 BEACON CRC trial [3], it is now possible to evaluate the potential of encorafenib (ENCO), binimetinib (BINI), and cetuximab (CET). In the BEACON CRC trial, progression-free survival (PFS) was longer in the ENCO, BINI, and CET group than in the control group (4.3 vs 1.5 months) [3]; Based on these results, ENCO, BINI, and CET therapies were covered by insurance in Japan in 2020. In the BEACON CRC study, the incidence of grade 3 adverse events was 58%. In such cases, drug withdrawal or dose reduction is considered. Here, we report a case of grade 3 pancreatitis that emerged after treatment with ENCO, BINI, and CET in a patient with BRAF-mutant colorectal cancer. This patient showed a long-term response after dose reduction.

## Case presentation

**Figure 1:**
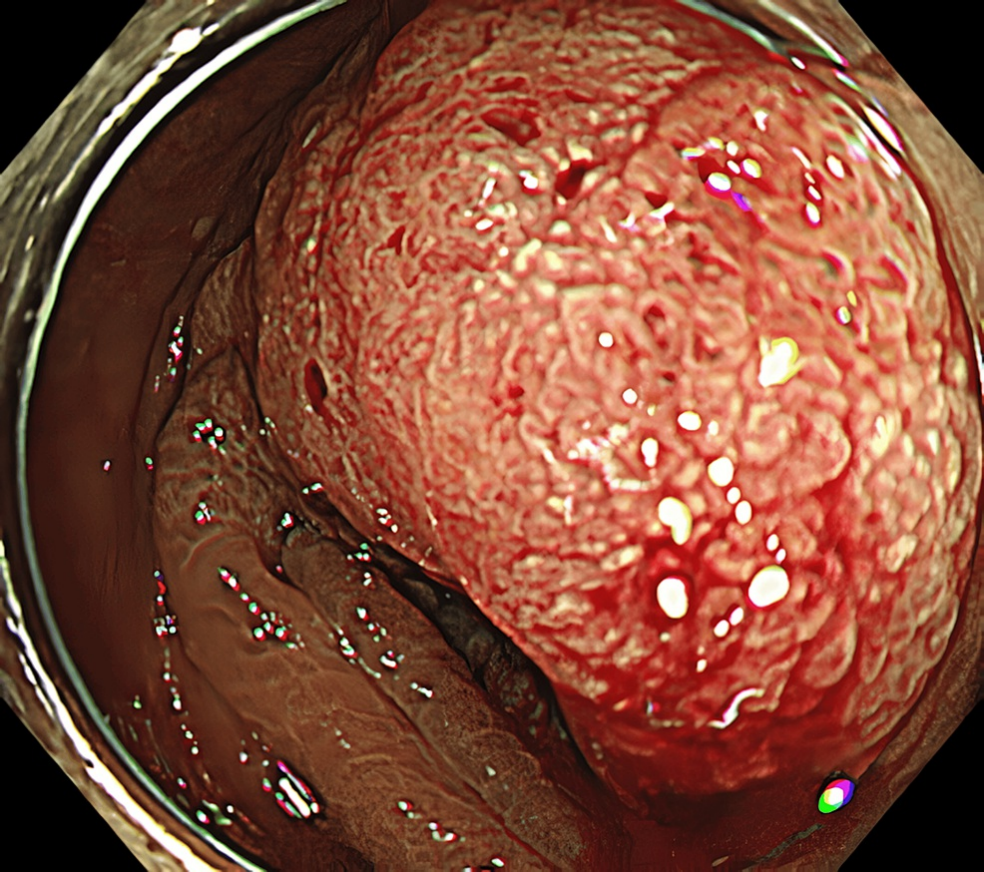
A semi-peripheral type 1 tumor is seen in the descending colon. The lumen is narrowed, and the scope passage is impossible.

**Figure 2:**
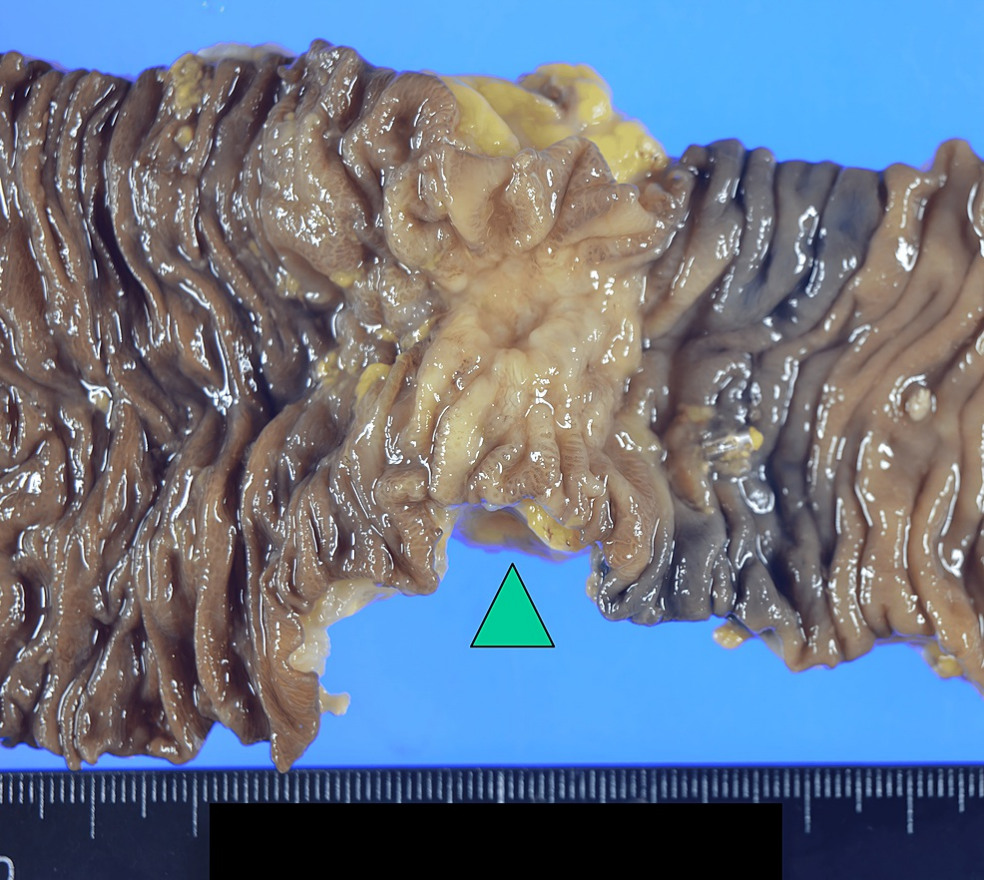
Macro image of the tumor.

The patient was a 74-year-old woman presenting with descending colon cancer. Prior to admission at our hospital, her colon had been found to be impassable via a scope (Figure 1), and she underwent laparoscopic resection of the left half of the colon. The intraoperative findings revealed seeded nodules throughout the peritoneum. Initial blood tests revealed the following: white blood cell count of 5,300 /µL, C-reactive protein (CRP) of 0.09 mg/dL, carcinoembryonic antigen (CEA) of 2.1 ng/mL, carbohydrate antigen (CA) 19-9 of 2,860 U/mL, and anti-p53 antibody of <0.40U/mL. The resected specimen shows the mass in the colon, measuring 28 mm in diameter (Figure 2). The pathological diagnosis was pT4a, pN2b, pM1c1 (P3), and pStage IVc (Japanese Classification of Colorectal, Appendiceal, and Anal Carcinoma, Ninth Edition). Genetic test results revealed that the patient was BRAF V600E mutation-positive, Rat sarcoma viral oncogene (RAS) mutation negative, and microsatellite stable.

**Figure 3:**
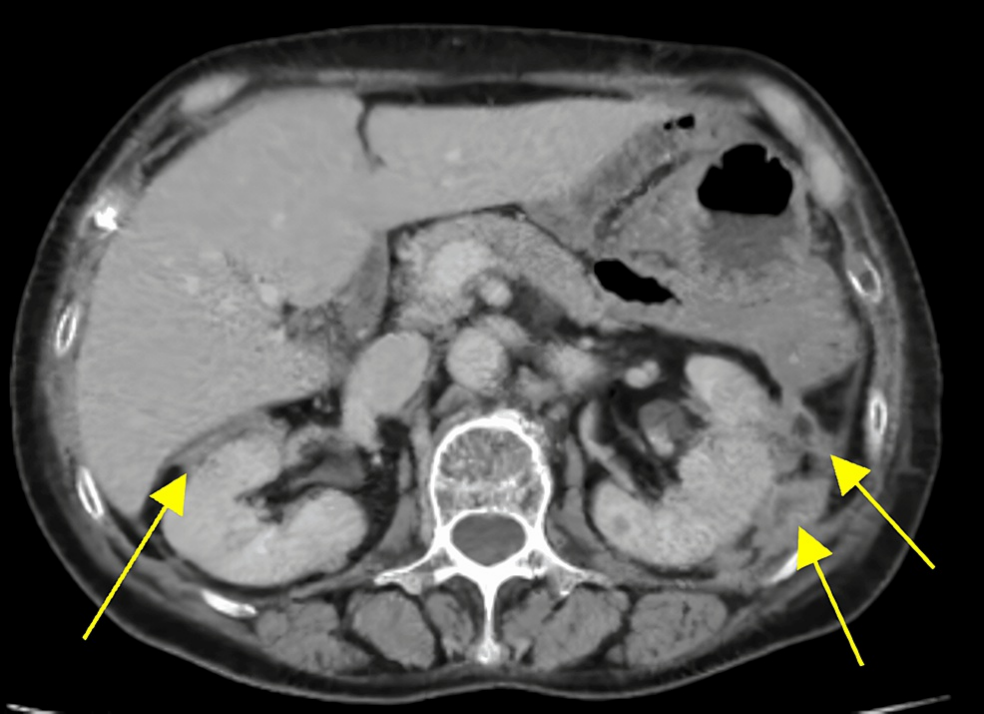
Computer tomography of a bilateral renal lesion prior to encorafenib, binimetinib, and cetuximab treatment.

After resection of the left colon, IRIS (irinotecan plus tegafur, gimestat, and otastat potassium) plus bevacizumab (BEV) was initiated as postoperative chemotherapy with a relative dose intensity of approximately 50% owing to poor tolerability. Nine months after the start of primary treatment, the patient developed enlarged disseminated nodules around both kidneys (Figure 3), and CET+ENCO+BINI was initiated.

**Figure 4:**
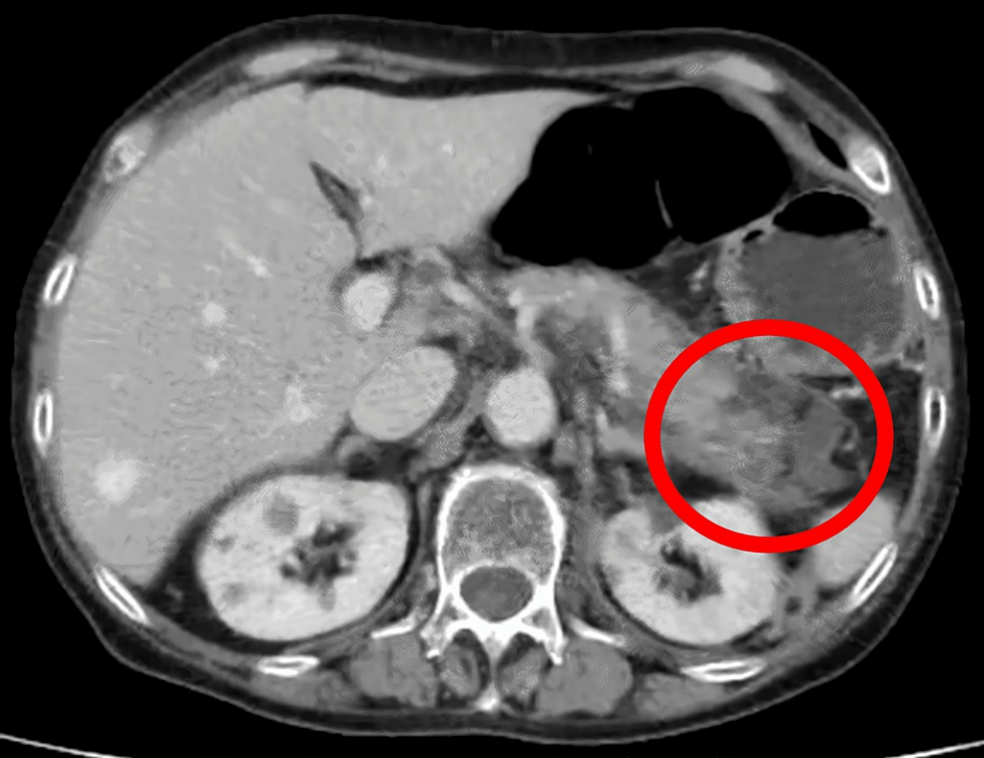
Pancreatic swelling, peripancreatic fatty opacity, and fluid retention appeared, which were findings of acute pancreatitis.

**Figure 5:**
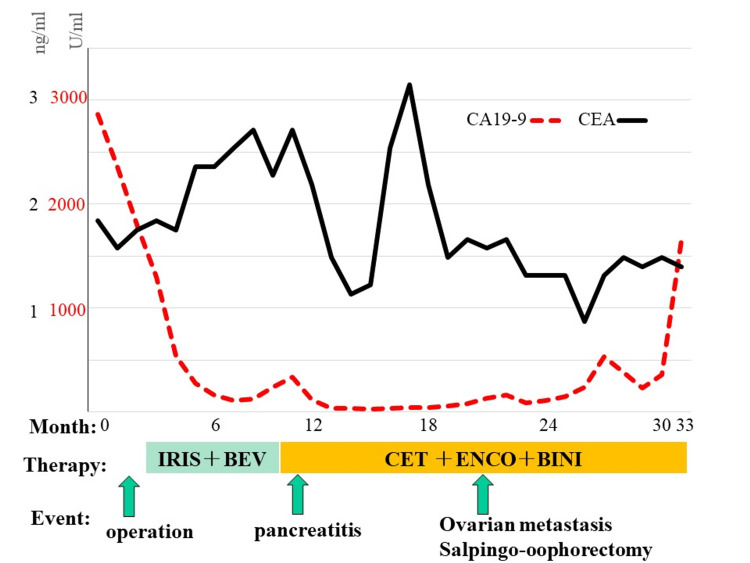
Tumor marker transition. CA 19-9, carbohydrate antigen 19-9; CEA, carcinoembryonic antigen; IRIS, irinotecan plus tegafur, gimestat, and otastat potassium; BEV, bevacizumab; ENCO, encorafenib, BINI, binimetinib, CET, cetuximab

**Figure 6:**
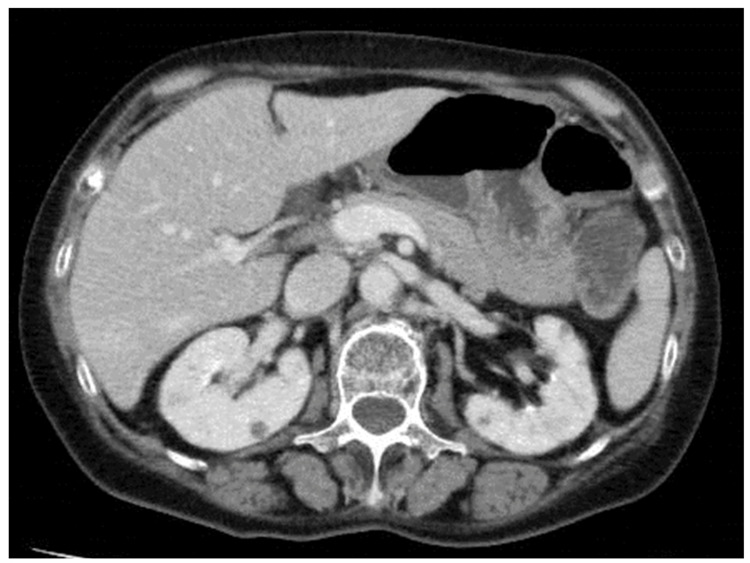
Computed tomography of the shrinking lesion in the abdomen seven months after the initiation of encorafenib, binimetinib, and cetuximab treatment.

One year after the first surgery and one month after the start of CET+ENCO+BINI, the patient visited the outpatient clinic with tenderness in the upper abdomen. Blood samples showed elevated amylase levels, and computed tomography (CT) showed an enlarged pancreas, mainly in the pancreatic body and surrounding fatty tissue opacity, and fluid retention (Figure 4). This led to the diagnosis of acute pancreatitis [4]. Blood samples obtained at admission revealed the following: amylase of 300 U/L, CRP of 16.75 mg/dL, blood urea nitrogen of 7 mg/dL, creatinine of 0.53 mg/dL, lactate dehydrogenase of 217 U/L, and platelet count of 346,000/µL. CT showed no inflammation above the level of the subrenal pole and no areas of poor contrast in the parenchyma. After admission, the patient was started on urinastatin 5MUq8. After the start of treatment, the abdominal pain improved and amylase levels normalized to 66 U/L. Ulinastatin was administered for five days and then discontinued. The day after urinastatin administration was completed, the dose of encorafenib was reduced from 300 mg to 225 mg and that of binimetinib was reduced from 90 mg to 60 mg. Treatment was resumed after consultation with the Department of Biliary and Pancreatic Endocrinology. Since then, no adverse events of grade 3 or higher have been reported. Tumor marker levels decreased (Figure 5), and both perineal metastases had almost disappeared (Figure 6) seven months after the initiation of CET+ENCO+BINI. After approximately 11 months of treatment, bilateral ovarian metastases appeared, and treatment was resumed with laparoscopic resection of the bilateral adnexa.

## Discussion

BRAF mutations are found in approximately 8% of patients with advanced recurrent colorectal cancer, often resulting in rapid progression and poor prognosis [5-8]. RAF is a kinase that constitutes the RAS-RAF-MEK-ERK (MAPK) intracellular signal transduction pathway. Signaling from RAS, which is activated by receptor-type tyrosine kinases such as epidermal growth factor receptor (EGFR), activates RAF, which, in turn, activates the downstream MEK-ERK pathway. It is believed that mutant BRAF permanently activates downstream MEK and ERK, with or without the activation of upstream RAS. Excessive signaling is believed to cause malignant transformation and tumor growth. Therefore, a three-drug combination therapy consisting of ENCO (a BRAF inhibitor), BINI (an MEK inhibitor), and CET (an anti-EGFR inhibitor) is considered effective [9,10]. The algorithm of the colorectal cancer treatment guidelines recommends CET, ENCO, or BINI as second- or third-line therapy after selecting the optimal regimen for first-line treatment based on the results of the BEACON CRC trial [11]. Adverse events including eye disorders, hepatic dysfunction, cardiac dysfunction, skin disorders, gastrointestinal disorders, and bleeding have also been reported according to drug information. Pancreatitis is another side effect of ENCO and BINI, but it is less frequent. In the BEACON CRC study, grade 3 adverse events occurred in more than half of the patients, and in this case, grade 3 pancreatitis developed. The total score of the Naranjo scale, which is a scale used to evaluate adverse drug reactions [12], was 7, indicating a high possibility of adverse drug reactions due to ENCO and BINI. After five days of treatment of pancreatitis, ENCO and BINI were re-initiated at reduced doses. Although the number of reports was small, the PFS was 11.7 months, which was longer than that of the BEACON CRC study (4.3 months) as a result of treatment of adverse events, appropriate timing of drug restart, and the ability to continue treatment with reduced drug doses. The dose should be carefully considered, especially in elderly patients with physiological impairments related to drug metabolism. It is known that BRAF mutation-positive colorectal cancer is frequently associated with microsatelite instability (MSI)-high. There have been reports of cases in which immune checkpoint inhibitors were successful; the use of immune checkpoint inhibitors as primary therapy may be considered in BRAF mutation-positive patients with MSI-high [13,14]. As this report is based on a single case, it is difficult to evaluate the clinical impact of ENCO, BINI, and CET therapy. Further case series and studies are required to determine the appropriate use and precautions for ENCO, BINI, and CET therapy in clinical practice.

## Conclusions

We have reported a case of grade 3 pancreatitis in a patient with BRAF V600E mutation-positive colorectal cancer treated with ENCO, BINI, and CET. However, the patient was able to continue treatment with appropriate pancreatitis therapy and drug reduction. In addition, the best treatment effect was partial response, with a PFS of 11.7 months, which was not inferior to the results of the BEACON CRC study. Though the number of cases is small, ENCO, BINI, and CET may be a regimen that can be administered to patients aged 70 years and older with appropriate dose reductions with attention to side effects. The details of the pathogenesis of pancreatitis caused by the use of BINI and ENCO are unknown, but it is hoped that further accumulation of cases will clarify this issue in the future.
